# Supplementary material for: Meta-Analysis of the Mechanisms Underlying COVID-19 Modulation of Parkinson’s Disease
Source: Int J Mol Sci. 2023 Aug 31;24(17):13554. doi: 10.3390/ijms241713554 (PMC10487929; doi:10.3390/ijms241713554)
Supplement: Supplementary file 1 [file ijms-24-13554-s001.zip › ijms-2509649-supplementary.pdf]

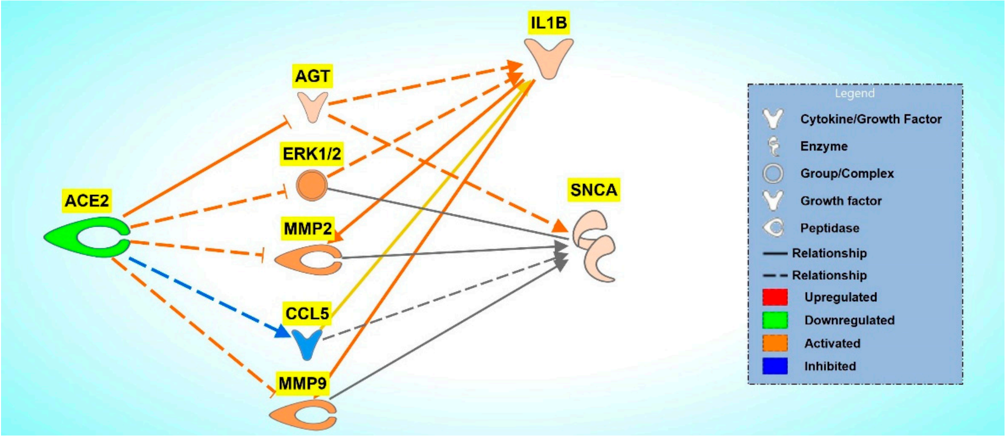

Figure S1a. Pathway depicting the downregulation of ACE2 affecting a set of grown molecules associated with IL1B (AGT, ERK1/2, MMP2, CCL5, MMP9) leading to the activation of SNCA.

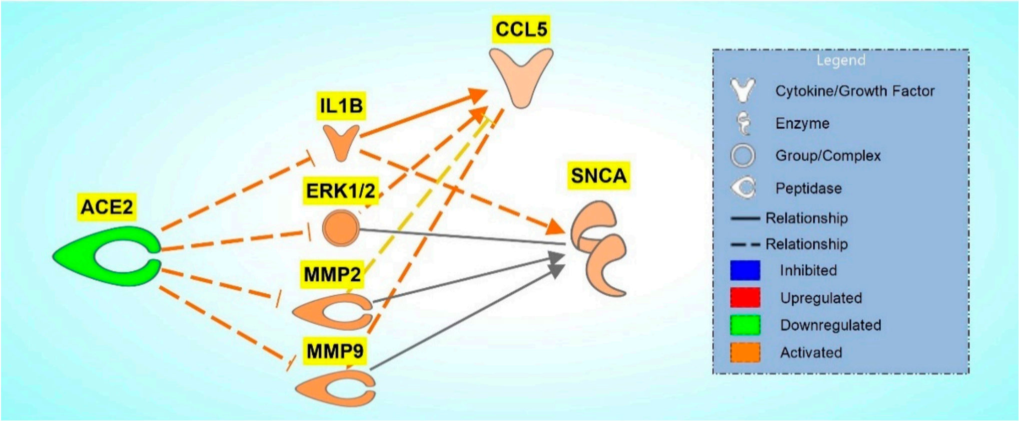

Figure S1b. Pathway depicting the downregulation of ACE2 affecting a set of grown molecules associated with CCL5 (IL1B, ERK1/2, MMP2, MMP9) leading to the activation of SNCA.

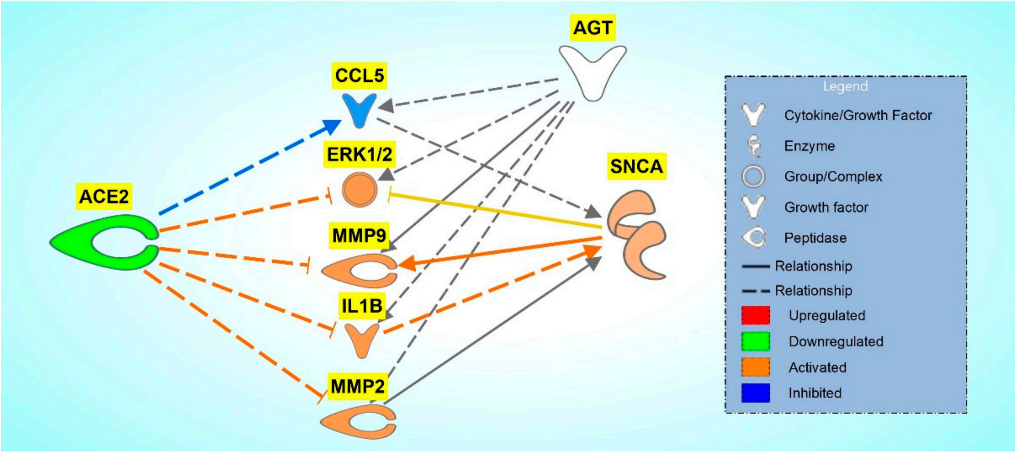

Figure S1c. Pathway depicting the downregulation of ACE2 affecting a set of grown molecules associated with AGT (CCL5, ERK 1/2, MMP9, IL1B, MMP2) leading to the activation of SNCA.

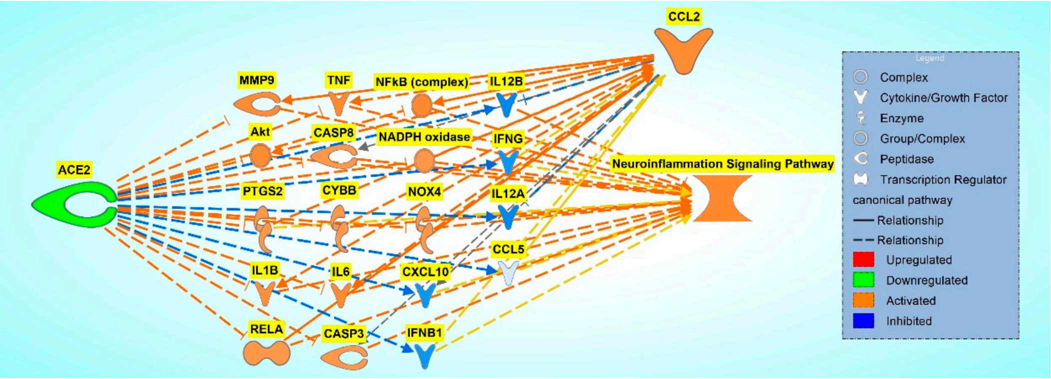

Figure S2a. Pathway depicting the downregulation of ACE2 affecting a set of grown molecules associated with CCL2 (MMP9, TNF, NFkB (complex), IL2B, Akt, CASP8, NADPH oxidase, IFNG, PTGS2, CYBB, NOX4, IL2A, IL1B, IL6, CXCL10, CCL5, RELA, CASP3, IFNB1) leading to the activation of the NISP.

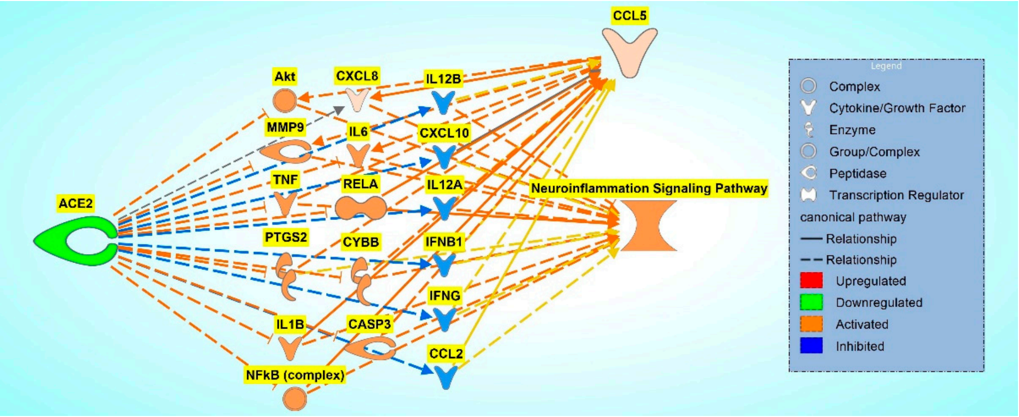

Figure S2b. Pathway depicting the downregulation of ACE2 affecting a set of grown molecules associated with CCL5 (Akt, CXCL8, IL2B, MMP9, IL6, CXCL10, TNF, RELA, IL2A, PTGS2, CYBB, IFNB1, IL1B, CASP3, CCL2, NFkB (complex)) leading to the activation of the NISP.

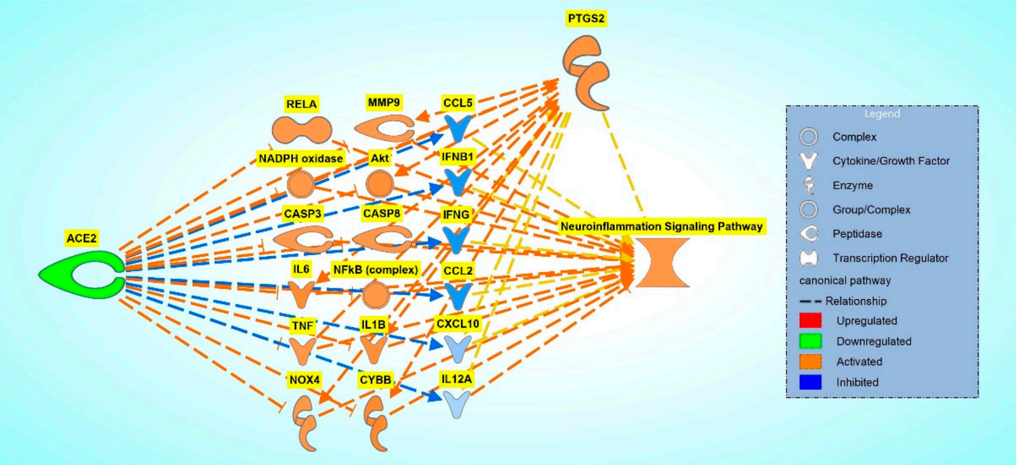

Figure S2c. Pathway depicting the downregulation of ACE2 affecting a set of grown molecules associated with PTGS2 (RELA, MMP9, CCL5, NADPH oxidase, Akt, IFNB1, CASP3, CASP8, IFNG, ILB, NFkB (complex), CCL2, TNG, IL1B, CXCL10, NOX4, CYBB, IL12A) leading to the activation of the NISP.

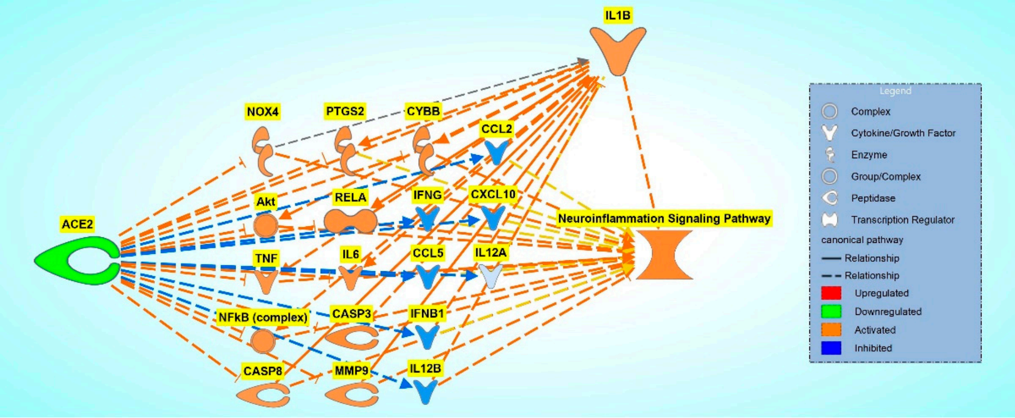

Figure S2d. Pathway depicting the downregulation of ACE2 affecting a set of grown molecules associated with IL1B (NOX4, PTGS2, CYBB, CCL2, Akt, RELA, IFNG, CXCL10, TNF, IL6, CCL5, IL12A, NFkB (complex), CASP3, IFNB1, CASP8, MMP9, IL12B) leading to the activation of the NISP.

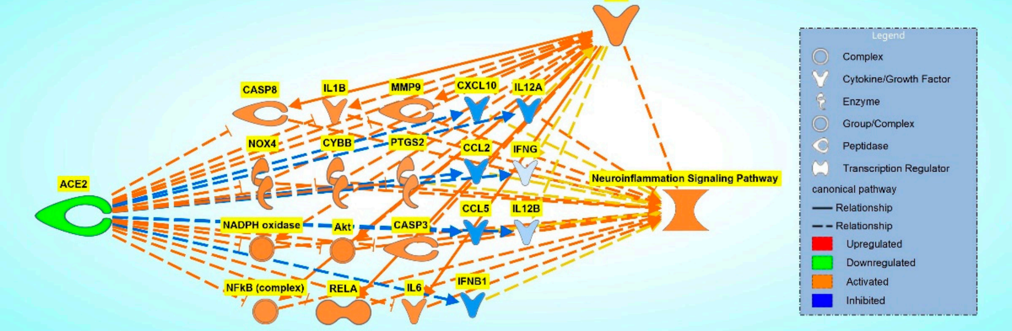

Figure S2e. Pathway depicting the downregulation of ACE2 affecting a set of grown molecules associated with TNF (CASP8, IL1B, MMP9, CXCL10, IL12A, NOX4, CYBB, PTGS2, CCL2, IFNG, NADPH oxidase, AKT, CASP3, CCL5, IL12B, NFkB (complex), RELA, IL6, IFNB1) leading to the activation of the NISP.

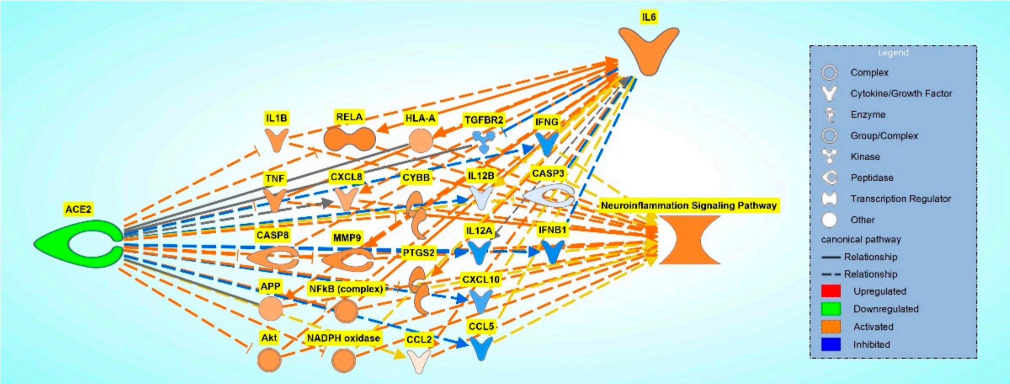

Figure S2f. Pathway depicting the downregulation of ACE2 affecting a set of grown molecules associated with IL6 (1ILB, RELA, HLA-A, TGFBR2, IFNG, TNF, CXCL8, CYBB, IL12B, CASP3, CASP8, MMP9, PTGS2, IL2A, IFNB1, APP, NFkB (complex), CXCL10, Akt, NADPH oxidase, CCL2, CCL5) leading to the activation of the NISP.
